# Ironing out the details: ferroptosis and its relevance to diabetic cardiomyopathy

**DOI:** 10.1152/ajpregu.00117.2023

**Published:** 2023-09-25

**Authors:** Flobater I. Gawargi, Paras K. Mishra

**Affiliations:** Department of Cellular and Integrative Physiology, University of Nebraska Medical Center, Omaha, Nebraska, United States

**Keywords:** cell death, diabetes, FSP1, heart, iron, LIP, lipid peroxidation, mitochondria, ROS

## Abstract

Ferroptosis is a newly identified myocardial cell death mechanism driven by iron-dependent lipid peroxidation. The presence of elevated intramyocardial lipid levels and excessive iron in patients with diabetes suggest a predominant role of ferroptosis in diabetic cardiomyopathy. As myocardial cell death is a precursor of heart failure, and intensive glycemic control cannot abate the increased risk of heart failure in patients with diabetes, targeting myocardial cell death via ferroptosis is a promising therapeutic avenue to prevent and/or treat diabetic cardiomyopathy. This review provides updated and comprehensive molecular mechanisms underpinning ferroptosis, clarifies several misconceptions about ferroptosis, emphasizes the importance of ferroptosis in diabetes-induced myocardial cell death, and offers valuable approaches to evaluate and target ferroptosis in the diabetic heart. Furthermore, basic concepts and ideas presented in this review, including glutathione peroxidase-4-independent and mitochondrial mechanisms of ferroptosis, are also important for investigating ferroptosis in other diabetic organs, as well as nondiabetic and metabolically compromised hearts.

## Graphical abstract

**Figure:**
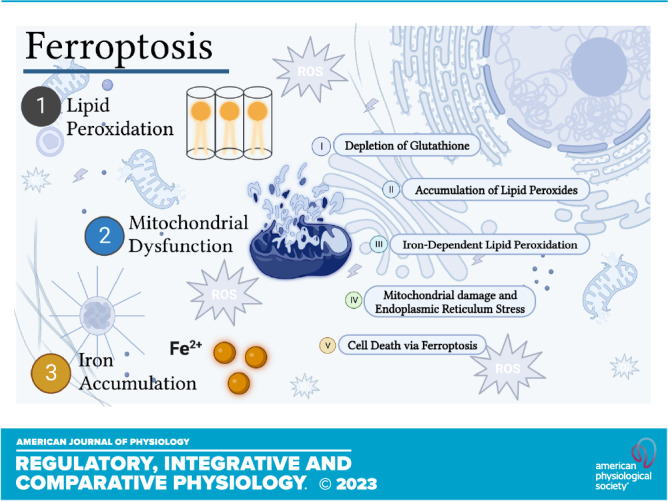

## INTRODUCTION

Nearly a decade ago, ferroptosis was discovered as a distinct iron-dependent mechanism of cell death driven by lipid peroxidation (1, 2). Ferroptosis is implicated in several diseases, such as cancer, neurodegeneration, kidney failure, liver disease, and cardiovascular disease (3–7). It is also involved in the death of pancreatic beta cells, causing diabetes and diabetic complications (7–9). However, very little is known about molecular mechanisms underpinning ferroptosis in diabetic cardiomyopathy (DMCM).

Death of cardiomyocytes, a terminally differentiated cell type with limited regenerative capacity in the adult heart, causes irreparable loss and instigates adverse cardiac remodeling leading to heart failure (10, 11). Out of multiple forms of cell death, at least six forms have been described in the heart (12, 13). Ferroptosis is one of the six forms of myocardial cell death (13) (Table 1). The molecular mechanisms underlying ferroptosis are poorly understood in DMCM.

**Table 1.** Overview of myocardial cell death mechanisms and their roles in diabetic cardiomyopathy

Type of Cell Death	Subtypes	Key Features	Key Regulators	Signaling Pathways	Roles in Diabetic Cardiomyopathy	PMID
Apoptosis	Intrinsic Extrinsic	Programmed Highly regulated, often neighboring cells remains unaffected Cell shrinkage Chromatin condensation DNA fragmentation Apoptotic bodies formation	Caspase-3 Caspase-9 Bcl2 Bax Calcium level pH	Mitochondrial dysfunction Death receptor signaling Endoplasmic reticulum stress Caspase signaling	Cardiomyocytes death Cardiac adverse remodeling Contractile dysfunction	32961025, 17562483, 32116717, 17599497, 34522203, 31131539, 30292723, 31418596
Mitochondrial-mediated necrosis	Not applicable	Cell swelling Membrane rupture Inflammation Loss of ATP production	Cyclophilin-D ANT VDAC	Ischemia Oxidative stress Calcium overload	Not reported	17599497, 11110769, 18345011, 17417626, 17694179, 31418596
Autophagy	Macro- Micro- Chaperone-mediated	Formation of autophagosomes Degradation of cellular components Nutrient recycling	LC3 Beclin-1 Atg5 Atg7	mTOR AMPK Insulin signaling Lysosomal signaling	Cardiac dysfunction in T1DM Cardioprotective in T2DM	21385586, 26042865, 33838320, 34522203, 30786833, 31418596
Pyroptosis	Caspase-1 dependent Caspase-4/5/11 dependent	Cell swelling Membrane pores Release of proinflammatory cytokines	GSDMD Caspase-1 NLRP3	Inflammasome activation IL-1β signaling IL-18 signaling	Induces inflammation Cardiac adverse remodeling Exacerbating diabetic cardiomyopathy	35145423, 31182921, 35538059, 25136835, 31418596
Necroptosis	Not applicable	Cell swelling Cell membrane rupture	RIPK1 RIPK3 MLKL	TNFα signaling Inflammatory	Contributes to inflammation Contributes to scar formation	32770173, 34194608, 34497836, 31418596
Ferroptosis	Not applicable	Rounded morphology Cytosolic vacuolization Cell membrane may disrupt Reduce mitochondrial size Increased mitochondrial density	GPX4 ACSL4 LIP	Iron homeostasis GPX4 signaling Lipid peroxidation signaling	Oxidative stress Increases myocardial cell Death Cardiac dysfunction	34258295, 35256941, 36313744, 31418596

ACSL4, Acyl-CoA synthetase long-chain family member 4; AMPK, adenosine monophosphate-activated protein kinase; ANT, adenine nucleotide translocator; Atg5, autophagy-related 5; Atg7, autophagy-related 7; Bax, Bcl-2-associated X protein; Bcl2, B-cell lymphoma 2; Beclin-1, beclin 1; Caspase-1, cysteine-aspartic acid protease 1; Caspase-3, cysteine-aspartic acid protease 3; Caspase-9, cysteine-aspartic acid protease 9; Cyclophilin-D, cyclophilin D; GPX4, glutathione peroxidase 4; GSDMD, gasdermin D; IL-1β, interleukin-1 β; IL-18, interleukin-18; LC3, microtubule-associated proteins 1A/1B light chain 3B; LIP, labile iron pool; MLKL, mixed lineage kinase domain-like protein; mTOR, mammalian target of rapamycin; NLRP3, NOD-, LRR- and pyrin domain-containing protein 3; RIPK1, receptor-interacting protein kinase 1; RIPK3, receptor-interacting protein kinase 3; TNFα, tumor necrosis factor α; T1DM, type 1 diabetes mellitus; T2DM, type 2 diabetes mellitus; VDAC, voltage-dependent anion channel.

Diabetes mellitus (DM) induces cardiac muscle disorder independent of hypertension, valvular disease, and coronary artery disease, leading to DMCM (14). DMCM increases the risk of heart failure, which is not abated by intensive glycemic control in patients with diabetes (15, 16). Thus, DMCM pathogenesis leading to heart failure is beyond hyperglycemia (17). Notably, there is no cure for DMCM-associated heart failure. Since intramyocardial lipid accumulation instigates heart failure in patients with diabetes and cell death is the precursor for DMCM, targeting ferroptotic cell death is a promising avenue to prevent and/or treat DMCM-associated heart failure (18–20).

In this review, we have elaborated on the mechanisms underpinning ferroptosis, provided best practices and technical approaches to evaluate ferroptosis, emphasized the importance of ferroptosis in myocardial cell death, and included key approaches to target ferroptosis in DMCM. We have also clarified several misconceptions regarding ferroptosis. Although this review is focused on DMCM, the basic concepts and ideas furnished here presented in this review are useful for investigating ferroptosis in other diabetic organs, including the kidney and the liver, as well as nondiabetic and metabolically compromised hearts.

## FERROPTOSIS IN DIABETIC CARDIOMYOPATHY

Ferroptosis is defined as an iron-dependent cell death mechanism driven by lipid peroxidation (1). Notably, the concept of ferroptosis was developed on the basis of three fundamental knowledge focused on cysteine, pillars: cysteine and polyunsaturated fatty acid (PUFA) metabolism, glutathione peroxidase-4 (GPX4)-dependent inhibition of lipid peroxidation, and effects of the redox-active labile iron pool (LIP) on PUFA (1, 21–25). The recent screening of new compounds to investigate regulated cell death mechanisms provided supplemental knowledge on developing the concept of ferroptosis. For example, erastin (eradicator of RAS and small T antigen-expressing cells) and RSL3 (RAS-selective-lethal-3) induce, but GPX4 inhibits ferroptosis (2, 26–31). Ferroptosis is a distinct form of cell death, and the key features of ferroptosis are not present in any other forms of cell death, such as apoptosis, pyroptosis, and necroptosis (1, 2). The three distinct features of ferroptosis are iron dependency, redox imbalance through glutathione and GPX4, and lipid peroxidation (32).

Ferroptosis in the heart is a relatively new discovery and was first reported in the year 2018 (33–36). The heart relies on a steady energy supply from mitochondria, which in turn require iron as a cofactor for various molecules and enzymes (37, 38). Thus, iron is critical for mitochondrial function, and consequently, for heart function (39). However, excessive iron causes mitochondrial dysfunction and has detrimental effects on the heart (40–42). Iron acts as a catalyst for reactive oxygen species (ROS) production, and excess iron increases oxidative stress and mitochondrial dysfunction, progressing to cardiomyopathy (42). A major pathway for iron import in cardiomyocytes, the contractile cells of the heart, is via L-type calcium channels, and iron overload in cardiomyocytes causes cardiomyopathy (43). Thus, ferroptosis has a pivotal role in iron-overload cardiomyopathies. There are several studies demonstrating the role of ferroptosis in adverse cardiac remodeling and heart failure (44).

In the past few years, only a limited number of studies have explored the role of ferroptosis in the hearts affected by either type-1 or type-2 DM (9, 45) (Table 2). Iron overload is linked to diabetes-induced pathogenesis in humans (46–48). However, there is limited knowledge on the regulation of iron homeostasis and the pathogenic effects of iron deregulation on the diabetic heart. Recent studies show a direct role of nuclear factor erythroid 2-related factor 2 (Nrf2) signaling in iron storage and export in the diabetic heart. Impaired Nrf2 signaling reduces iron storage into ferritin in the streptozotocin-induced type 1 diabetes mellitus (T1DM) heart, while it downregulates ferroportin-1 compromising iron export in the high-fat diet and streptozotocin-induced T2DM heart (9, 45).

**Table 2.** Studies on diabetes-induced cardiac ferroptosis

T1DM				T2DM			
Model	Upregulated	Downregulated	PMID	Model	Upregulated	Downregulated	PMID
*In vivo studies*							
Rabbit (STZ)		NRF2, GPX4	35909950	Mouse STZ + HFD	MDA, LIP	GSH, GGSH/GSSG, SL7A11, Ferritin heavy chain	35256941
Rat (STZ)	ACSL4	GPX4	31809190	db/db	4-HNE, MDA, ACSL4, LIP	GPX4 GSH/GSSG, GSH, FSP1	36864033 36946337
Mouse (STZ)	ACSL4, NRF2, TFR1, LIP, MDA	GPX4, Ferroportin-1, GSH	36313744				

Model	Upregulated	Downregulated	PMID
*In vitro studies*			
Mouse neonatal cardiomyocytes	ACSL4, TFR1, NCOA4, LIP	GPX4	36946337
H9C2 cell line	NRF2, MDA, LIP, Ferroportin-1, TFR, ACSL4	GPX4, GSH	36624878 36313744 31809190

ACSL4, Acyl-CoA synthetase long-chain family member 4; 4-HNE, 4-hydroxynonenal; FSP1, ferroptosis suppressor protein 1 (also known as AIFM2-apoptosis-inducing factor mitochondrion-associated 2); GPX4, glutathione peroxidase 4; GSH, glutathione (reduced form); GSSG, glutathione disulfide (oxidized form of glutathione); LIP, labile iron pool; MDA, malondialdehyde; NCOA4, nuclear receptor coactivator 4; NRF2, nuclear factor erythroid 2-related factor 2; TFR1, transferrin receptor 1; SLC7A11, solute carrier family 7 member 11; STZ, streptozotocin; HFD, high-fat diet.

Further studies are warranted to explore the regulation of iron import, the conversion of imported iron into redox active LIP, and iron storage and export mechanisms. In addition, studies on the regulation of PUFA peroxidation and GPX4 expression as well as activity are needed to understand the molecular regulation of ferroptosis in DMCM. The mechanisms by which mitochondrial GPX4, mitochondrial iron, and metabolic imbalances contribute to ferroptosis remain nebulous in the heart and are unknown in the diabetic heart.

## PREDOMINANT ROLE OF FERROPTOSIS IN DIABETIC CARDIOMYOPATHY

**Figure 1.**
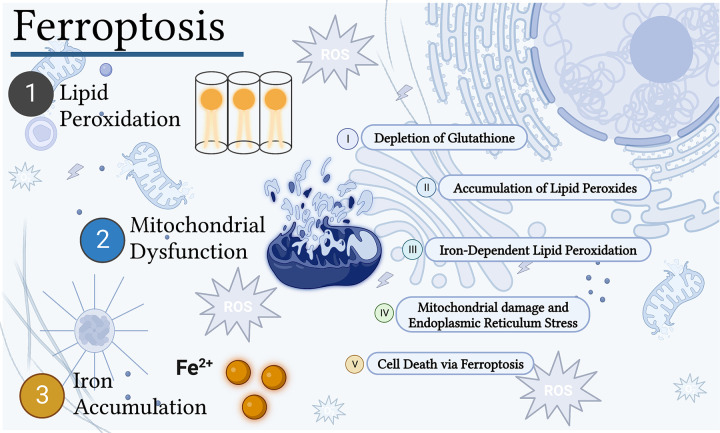
Predominant role of ferroptosis in diabetic cardiomyopathy. Increased lipid peroxidation (*1*), mitochondrial dysfunction (*2*), and iron accumulation (*3*) indicate a predominant role of ferroptosis in diabetic cardiomyopathy. The combined presence of polyunsaturated fatty acid (PUFA), iron [redox active labile iron pool (Fe^2+^)], and reactive oxygen species (ROS), due to mitochondrial dysfunction and endoplasmic reticulum (ER) stress, promote lipid peroxidation in the diabetic heart. Depletion of glutathione impairs PUFA-ROS reducing activity of glutathione peroxidase 4 (GPX4) and exacerbates lipid peroxidation in the diabetic heart, which leads to ferroptotic cell death. Figure created with BioRender.com.

Ferroptosis may play a predominant role in DMCM due to several reasons. First, lipid peroxidation is a key feature of ferroptosis, and intramyocardial lipid accumulation is augmented and is directly linked to the instigation and exacerbation of cardiac pathogenesis in patients with diabetes (2, 18). Second, aberrant metabolism and dysfunctional mitochondria independently induce ferroptosis, and both are hallmarks of the diabetic heart (49–51). Third, iron is indispensable for ferroptosis, and circulating iron levels are elevated in patients with diabetes (47). Fourth, the combined presence of the three key major components of ferroptosis (excess lipid, oxidative radicals, and iron) points to a predominant role of ferroptosis in DMCM (Fig. 1). Nevertheless, there is limited knowledge of molecular mechanisms and regulatory pathways involved in DM-induced cardiac ferroptosis.

## EVALUATING FERROPTOSIS IN DIABETIC CARDIOMYOPATHY

Ferroptosis is a unique mechanism of cell death, which is distinct from apoptosis, necrosis, and autophagy. Ferroptotic cells do not exhibit apoptotic bodies, cell shrinkage, and chromatin condensation—the key features of apoptosis; cytoplasmic swelling and cell membrane rupture—the key features of necrosis; and autophagosome formation—a key feature of autophagy (52). These cells also do not display features of pyroptosis or necroptosis (13). Ferroptotic cells typically display a rounded morphology and are characterized by cytosolic vacuolization (53). There are at least six forms of cell death in the heart, including ferroptosis (Table 1). We have elaborated the best practices to evaluate myocardial cell death, including ferroptosis, in the “Guidelines for evaluating myocardial cell death” (13). In brief, the characteristic features of ferroptosis are reduced GPX4 and glutathione, increased levels of lipid peroxides, such as 4-hydroxynonenal (4-HNE) and malonaldehyde (MDA), and increased redox-active labile iron pool (LIP). The additional features include a decrease in lipid peroxide levels by treatment with ferroptosis inhibitor ferrostatin-1, reduced mitochondrial cristae size, and smaller mitochondria (13). In addition, evaluating ferroptosis and understanding its molecular mechanisms in DMCM requires attention to the upregulation of molecules involved in lipid peroxidation and the downregulation of those that prevent it. An updated approach for ferroptosis evaluation in the diabetic heart is described in Table 3.

**Table 3.** Assessment of ferroptosis in the diabetic heart

Molecules	Mechanism	Measurement (PMID)	Expression in Ferroptosis and Comments
GPX4 expression	Required for reducing lipid-ROS	Western blotting (25922076); Immunohistochemistry (36443446)	Reduce. A key marker of ferroptosis. Express in cytoplasm and mitochondria.
GPX4 activity	Converts lipid-ROS into lipid alcohol by oxidizing glutathione (GSH), which converts into GSSG (reduced form)	Calorimetry assay (31418596) using commercial kit	Reduce. A key marker of ferroptosis. Activity is quick and thus time sensitive.
Lipid peroxides	Produced by lipid peroxidation. 4-hydroxynonenal (4-HNE), a hydroxyalkenal, and malondialdehyde (MDA), a major aldehyde derived from lipid peroxidation, are produced by ferroptosis.	Western blotting (4-HNE, anti-MDA adduct 1F83), calorimetric assay using commercial kit. Thiobarbituric acid reactive substance (TBARs) assay (11483619)	Increase. A key marker of ferroptosis. TBARs is a novel assay kit to measure MDA in biological samples, including fluids.
LIP	Oxidizes lipid into lipid peroxides	Calorimetric assay (35256941)	Increase. Produced by Fenton reaction and/or by ferritinophagy.
Glutathione disulfide reductase (GSR)	GSR oxidizes GSSG into GSH, which is used for GPX4 activity	Western blotting	Reduced.
ACSL4	Activates PUFA. A key molecule for the execution of ferroptosis.	Western blotting	Increase. A putative biomarker of ferroptosis.
LPCAT3	Adds phospholipid to PUFA. A key molecule for the membrane localization of PUFA.	Western blotting	Increase.
PKCβII	Activates ACSL4	Western blotting	Increase. A key sensor of status of ferroptosis.
Ferroportin-1	Efflux cellular iron	Western blotting	Decrease. A major protein for iron export
Hepcidin	Binds to ferroportin-1 to block iron efflux	Western blotting	Decrease.
Ferritin heavy chain (FHC)	Iron storage in the form of ferritin	Western blotting	Decrease. Downregulates LIP.
Ferritin light chain (FLC)	Facilitate iron storage in ferritin	Western blotting	Decrease. Downregulates LIP.
Poly(rc)-binding protein 1 (PCBP1)	Regulate iron storage in ferritin	Western blotting	Decrease. Downregulates LIP.
Nuclear receptor coactivator 4 (NCOA4)	Induces ferritinophagy to increase the levels of LIP	Western blotting	Increase. Upregulates LIP.
Transferrin and transferrin receptor 1	Import iron into cells	Western blotting	Increase.
Ferroptosis Suppressor Protein 1 (FSP1)	Similar to GPX4 that uses GSH to reduce lipid-ROS, FSP1 uses NADHP to reduce Coenzyme Q_10_.	Western blotting	Decrease.
Mitochondrial morphology	Disappearance of cristae, outer membrane disruption, volume reduction	Electron microscopy (29024608)	Increase.
Cellular morphology	Ballooning phenotype recognized by the formation of a clear, rounded cell with empty cytosol	Electron microscopy (32575749)	Increase.

ACSL4, Acyl-CoA synthetase long-chain family member 4; GPX4, glutathione peroxidase 4; LIP, labile iron pool; LPCAT3, lysophosphatidylcholine acyltransferase-3; PKCβII, protein kinase c β-type isoform-II; PUFA, polyunsaturated fatty acid; ROS, reactive oxygen species.

## UNEXPLORED MECHANISMS OF FERROPTOSIS IN DIABETIC CARDIOMYOPATHY

**Figure 2.**
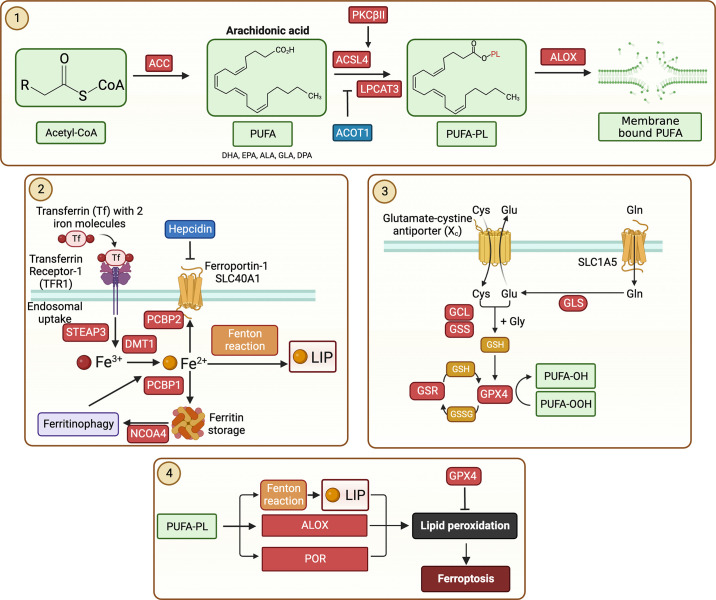
Potential mechanisms of ferroptosis in diabetic cardiomyopathy. The mechanisms of 4 major steps in ferroptosis are shown with key molecules and regulatory proteins. In the first step, polyunsaturated fatty acid (PUFA, such as arachidonic acid) is produced from acetyl-coenzyme A (Acetyl-CoA) by the enzymatic action of acetyl-CoA carboxylase (ACC). PUFA is activated by the enzyme acetyl-CoA synthetase long-chain family member 4 (ACSL4), and phospholipid (PL) is added to the activated PUFA by the enzyme lysophosphatidylcholine acyltransferase 3 (LPCAT3). While acyl-CoA thioesterase-1 (ACOT1) inhibits, arachidonate lipoxygenase (ALOX) promotes PUFA-PL formation. Notably, all lipids may not lead to ferroptosis. In the second step, transferrin incorporating iron (ferric, Fe^3+^) is imported into the cell via transferrin receptor 1 (TFR1). The ferric iron is converted into ferrous iron (Fe^2+^) with the help of ferric reductase six-transmembrane epithelial antigen of prostate 3 (STEAP3) and divalent metal transporter 1 (DMT1). Ferrous iron undergoes Fenton reaction to produce labile iron pool (LIP), which causes peroxidation of PUFA-PL. LIP level is regulated by either storage of ferrous iron into ferritin or efflux of ferrous iron via ferroportin-1, which is facilitated by solute career family-48 member-1 (SLC48A1). Hepcidin blocks ferroportin-1 to export iron. Hepcidin is important for maintaining iron levels in the iron-deficient cell. In the third step, cystine is imported through the exchange of glutamate via system Xc^−^. It is reduced into cysteine in the cytoplasm. Cysteine and glutamate biosynthesize glutathione (GSH) in a two-step process by glutamate-cysteine ligase and subsequently glutathione synthetase (GSS) enzymes. Glutamate levels are maintained in the cell via its import through solute career family-1 member-A5 (SLC1A5). GSH is utilized by glutathione peroxidase 4 (GPX4) to reduce PUFA-ROS resulting in the oxidation of GSH to GSSG. Glutathione reductase (GSR) reduces GSSG to GSH and maintains the activity of GPX4. In the fourth step, PUFA-PL is converted into PUFA-peroxide by LIP, ALOX, and nicotinamide adenine dinucleotide phosphate (NADPH)-cytochrome p450 oxidoreductase (POR). Lipid peroxides cause ferroptotic cell death. Downregulation of GPX4, an inhibitor of lipid peroxide formation, promotes ferroptosis. ALA, α-linolenic acid; DHA, docosahexaenoic acid; EPA, eicosapentaenoic acid; GCL, glutamate cysteine ligase; GLA, gamma-linolenic acid; DPA, docosapentaenoic acid; NCOA4, nuclear receptor coactivator 4; PCBP1, poly(rc)-binding protein 1; PCBP2, poly(rc)-binding protein 2; PKCβII, protein kinase c β-type isoform-II; ROS, reactive oxygen species. Figure created with BioRender.com.

Diabetes is associated with aberrant lipid metabolism, and the excessive lipid buildup in the heart may enhance vulnerability of cardiomyocytes to ferroptosis (18, 54, 55). Ferroptosis can be broadly divided into four major steps: *1*) formation of PUFA-phospholipids (PUFA-PL), *2*) production of LIP, *3*) impaired biosynthesis and activation of GPX4, and *4*) production of lipid peroxides (Fig. 2). The mechanisms of these steps are described below. Future research is required to better understand mechanisms underpinning ferroptosis in DMCM.

### Formation of PUFA-PL

Lipid peroxidation predominantly occurs in PUFAs, which are especially susceptible due to their multiple carbon–carbon double bonds (C=C). During lipid peroxidation, the weak carbon–hydrogen (C–H) bonds located between these double bonds are replaced by a peroxyl (O–O) groups. For example, linoleic acid (18:2) is an 18-carbon PUFA with two such double bonds (1). Another example of PUFA is arachidonic acid (20:4), which has 20 carbons with 4-carbon double bonds. PUFA is synthesized by acetyl CoA with the help of the acetyl-CoA carboxylase enzyme. The synthesis of PUFA is inhibited by adenosine monophosphate-activated protein kinase (AMPK), the nutrient sensor of the cell (56, 57). PUFA is activated (oxidized) to drive ferroptosis. The activation of PUFA requires acyl-coenzyme A synthetase long-chain family member 4 (ACSL4) enzyme. Activated PUFA is incorporated into membrane lipids, such as phospholipids (PL), for peroxidation, leading to ferroptosis. Lysophosphatidylcholine acyltransferase 3 (LPCAT3) enzyme attaches PL to PUFA, producing PUFA-PL, which is a membrane-localized PUFA (58). Intact oxidized PUFA-PL is indispensable for the execution of ferroptosis, and cleavage of PL from PUFA-PL inhibits ferroptosis (59). There are different types of PL (>350 types), but only specific types of PL—PLs with two PUFA tails—are key drivers of ferroptosis (60). Acyl-CoA thioesterase-1 (ACOT1) prevents the formation of PUFA-PL and protects against ferroptosis (61). Arachidonate lipoxygenase (ALOX), an iron-containing enzyme, catalyzes the deoxygenation of PUFA-PL to produce PUFA hydroperoxides (62). However, the role of ALOX in driving ferroptosis is controversial due to its radical-trapping antioxidant activity (63).

ACSL4 is a key molecule for the execution of lipid peroxidation. The Merlin-Hippo-YAP pathway regulates ACSL4 to control sensitivity to ferroptosis (64). Depending on initial lipid peroxidation events, ACSL4 is activated (via phosphorylation at the Thr328) by protein kinase c β-type isoform-II (PKCβII). This activation facilitates the incorporation of more PUFAs into the membrane (PUFA-PL), thereby amplifying ferroptosis and ultimately leading to cell death (65). Thus, ACSL4 is a key executor of ferroptosis and may act as a biomarker of ferroptosis, and PKCβII is a key sensor for the status of ferroptosis in a cell.

Future investigations on mechanisms for upregulation and activation of PUFA in the diabetic heart may provide novel approaches to mitigate cardiac ferroptosis in DMCM.

### Production of LIP

As the name suggests, iron plays a pivotal role in ferroptosis, being both directly and indirectly involved in the process of lipid peroxidation. The iron-dependent (iron serves as a cofactor) arachidonate lipoxygenases (ALOXs) enzymes act on PUFA-PL to form their hydroperoxides (PUFA-PL-OOH), which are the direct substrate of LIP (Fe^2+^) for Fenton reaction (Fe^2+^ + HOOH → Fe^3+^ + OH**^−^** +OH˙) generating free radicals that react with PUFA-PL-OOH to produce PUFA peroxides (PUFA-PL-OO˙) (3, 62, 63, 66). Not all forms of iron are involved in ferroptosis. Only the redox-active LIP drives ferroptosis. The amount of LIP depends on the regulation of iron homeostasis (import, storage, and export) in a cell. Increased import and reduced export and/or storage of iron may increase LIP in a cell.

Iron is an essential nutrient that plays a critical role in several biological processes. Iron is necessary for the creation of heme, a component of hemoglobin in red blood cells and myoglobin in muscle cells, which facilitates the transfer and storage of oxygen. It is also a cofactor for enzymes involved in energy production, DNA synthesis, and other vital cellular functions. Disruption of iron homeostasis can lead to LIP generation (67). Iron is taken from the food in the small intestine, and its absorption, storage, and use are delicately regulated to maintain a homeostatic level. The liver plays a crucial role in iron metabolism, storing excess iron as ferritin and releasing it as needed. Transferrin is the principal iron-binding protein in the blood, and it transports iron to various tissues, where it contributes to multiple disorders (68).

Iron is imported into a cell as ferric iron (Fe^3+^) bound to transferrin (TF) by transferrin receptor-1 (TFR1) or via L-type calcium channels in cardiomyocytes (43, 69). Heat shock protein β-1 (HSPB1) blocks iron import to inhibit ferroptosis (70). Ferric iron is converted into ferrous iron (Fe^2+^) in the endosome by ferric reductase six-transmembrane epithelial antigen of prostate 3 (STEAP3) (20, 44). Ferrous iron is transported into the cytosol by divalent metal transporter 1 (DMT1) to form LIP (71). Tight regulation of the LIP is critical for not only cellular function but also minimizing the risk of initiating ferroptosis through the formation of lipid peroxides. The levels of LIP can be decreased either by its storage as ferritin with the help of poly(rc)-binding protein 1 (PCBP1) or by its extracellular export through ferroportin-1 (blocked by hepcidin) and prominin 2 (72–74). Notably, iron stored in ferritin can be used to increase the levels of LIP via ferritinophagy, where ferritin undergoes selective autophagy with the help of the nuclear receptor coactivator 4 (NCOA4) enzyme (75). Thus, key molecules regulating cellular iron homeostasis, and consequently ferroptosis, include those involved in iron import (TF and TFR1), conversion of imported iron into LIP (STEAP3 and DMT1), storage of LIP into ferritin (PCBP1), LIP export outside the cell (ferroportin-1, hepcidin, and prominin 2), and formation of LIP via ferritinophagy (NCOA4).

Future investigations on mechanisms of iron import, LIP formation, and export in different types and stages of DMCM will unravel how to target cardiac ferroptosis in DMCM.

### Impaired GPX4 Biosynthesis and Activation

Reduced GPX4 activity is a hallmark of cardiac ferroptosis (13). GPX4, a selenoprotein, reduces lipid hydroperoxides into alcohol and thus inhibits lipid peroxidation (76). For lipid antioxidant activity, GPX4 requires a glutathione (GSH) substrate. GSH is oxidized to GSSG by GPX4 to reduce lipid hydroperoxides. The oxidized GSSG is reduced to GSH by the enzyme glutathione reductase (GSR). Thus, GSR is a crucial enzyme for maintaining the availability of GSH in the reduced form, and consequently, for sustaining GPX4 activity. GSH is biosynthesized from cysteine and glutamate in a two-step reaction where glutamate-cysteine ligase is involved in the first step and glutathione synthetase (GSS) is involved in the second step (77). Cysteine is the reduced form of cystine. Cystine is imported into the cell with the exchange of glutamate via system X_C_^−^ (76). Erastin inhibits system X_C_^−^ to induce ferroptosis (2). Ras-selective lethal 3 (RSL3) directly inhibits GPX4 activity, thereby induce ferroptosis (78).

Additional mechanisms for reduced GPX4 activity are efflux of GSH through multidrug resistance gene MDR1 and depletion of cysteine and thereby GSH by catabolic enzyme cysteine dioxygenase 1 (CDO1) (79, 80). Besides decreased GPX4 activity, reduced GPX4 expression also induces ferroptosis. Ferroptosis inducer 56 (FIN56) and chaperone-mediated autophagy degrade GPX4 to drive ferroptosis (81, 82). Thus, signaling molecules that regulate GPX4 expression and activity have critical roles in the regulation of ferroptosis.

Future investigations on these mechanisms in DMCM will unravel the specific cause for reduced GPX4 expression and activity and elevated levels of lipid peroxides in different stages of DMCM.

### Production of Lipid Peroxides

PUFA-PL hydroperoxides are converted into lipid peroxides in the presence of LIP. Nicotinamide adenine dinucleotide phosphate (NADPH)-cytochrome p450 oxidoreductase (POR) is a key mediator of and fuel for ferroptosis (83, 84). Both POR and NADH-cytochrome b5 reductase (CYB5R1) generate hydrogen peroxide (H_2_O_2_) by transferring electrons from NAD(P)H to oxygen. H_2_O_2_ reacts with iron to generate hydroxyl radical (OH˙), which in turn reacts with lipid hydroperoxide (PUFA-PL-OOH) to generate lipid peroxide (PUFA-PL-OO˙) (85). Reduced GPX4 expression and/or activity facilitates the production of lipid peroxides because GPX4 is the key molecule that converts toxic lipid hydroperoxides into nontoxic lipid alcohols.

GPX4-independent mechanisms are also present to control lipid peroxidation. Coenzyme Q_10_ (CoQ_10_), also known as ubiquinone or vitamin Q_10_, is present in mitochondria and diverse cell membranes (86). CoQ_10_ reduces lipid peroxides independent of GPX4. While reducing lipid peroxide, CoQ_10_ itself becomes oxidized. Ferroptosis suppressor protein 1 (FSP1) uses NADPH to regenerate reduced CoQ_10_ (87, 88). Thus, FSP1 and CoQ_10_ act together to reduce lipid peroxides. The abundance of reduced CoQ_10_ increases due to remodeling of the lipid membrane environment by guanosine triphosphate cyclohydrolase 1 (GCH1). Like CoQ_10_, GCH1 produces endogenous metabolite tetrahydrobiopterin (BH4), a potent radical-trapping antioxidant that protects the lipid membrane from autooxidation, to reduce lipid peroxides (89, 90). Mitochondrial CoQ_10_ is reduced by dihydroorotate dehydrogenase (DHODH), which is analogous to cytosolic FSP1 (91). Indole-3-pyruvate (I3P) is a free radical scavenger derived from tryptophan by interleukin-4-induced 1 (IL4i1), which is an amino acid oxidase secreted from immune cells (92). IL4i1 reduces lipid peroxides independent of GPX4.

Further investigations on these mechanisms in DMCM will reveal the cause for increased lipid peroxidation and novel approaches to prevent and/or treat cardiac ferroptosis in DMCM.

## MITOCHONDRIAL MECHANISMS OF FERROPTOSIS

DMCM is associated with mitochondrial damage and dysfunction, and improving mitochondrial health has the potential to mitigate DMCM (93, 94). Mitochondria is considered as a multifaceted regulator of cell death (95). In ferroptosis, both iron homeostasis in mitochondria and the presence of the mitochondrial form of GPX4 (mitoGPX4) serve as protective factors. A disruption or mitigation in these factors can lead to lipid peroxidation in the mitochondrial membrane, ultimately resulting in mitochondrial damage and triggering ferroptosis (50, 96).

Mitochondria contains 20%–50% of cellular iron and is a hub of iron homeostasis (97, 98). Although mitochondrial iron has a beneficial role in the production of heme and iron-sulfur (Fe-S) clusters, redox-active LIP is also present in mitochondria to induce lipid peroxidation that drives ferroptosis (99, 100). Iron homeostasis in mitochondria involves iron import, its storage in the form of mitochondrial ferritin, and its export. Iron (Fe^2+^) is imported into mitochondria via mitoferrin 2 (ubiquitous) or mitoferrin 1 (erythroid lineage) (101). This increases the levels of mitochondrial LIP, which augments mitochondrial ROS production, leading to mitochondrial membrane lipid peroxidation and ferroptosis. Mitochondrial outer membrane protein mitoNEET, also known as CDGSH iron-sulfur domain-containing protein-1 (CISD1), prevents iron import into mitochondria and thereby prevents mitochondrial lipid peroxidation (102). Mitochondrial LIP is stored in the mitochondria-specific form of ferritin (FTMT) to prevent the increase in mitochondrial ROS levels. Mutation in the mitochondrial ferritin encoding gene causes greater retention of iron in mitochondria, resulting in mitochondrial iron overload, which consequently reduces cytoplasmic iron levels (103). Mitochondrial iron export is regulated by the ATP-binding cassette (ABC) transporter 8 (ABCB8), which is present in the inner mitochondrial membrane. Targeted deletion of ABCB8 in mouse hearts causes decreased mitochondrial iron export, increased mitochondrial iron accumulation, and cardiomyopathy (104). Iron is used in the biosynthesis of heme in mitochondria, which has a crucial role as a cofactor in enzymatic reactions and electron transport (105). ABCB10 is initially thought to export iron from mitochondria; however, studies on neonatal rat cardiomyocytes demonstrated that ABCB10 is not a heme exporter, rather, it regulates heme biosynthesis in mitochondria (106). Feline leukemia virus subgroup C receptor 1 b (FLVCR1b) is also involved in the regulation of mitochondrial iron export via the efflux of mitochondrial heme into the cytoplasm (107).

**Figure 3.**
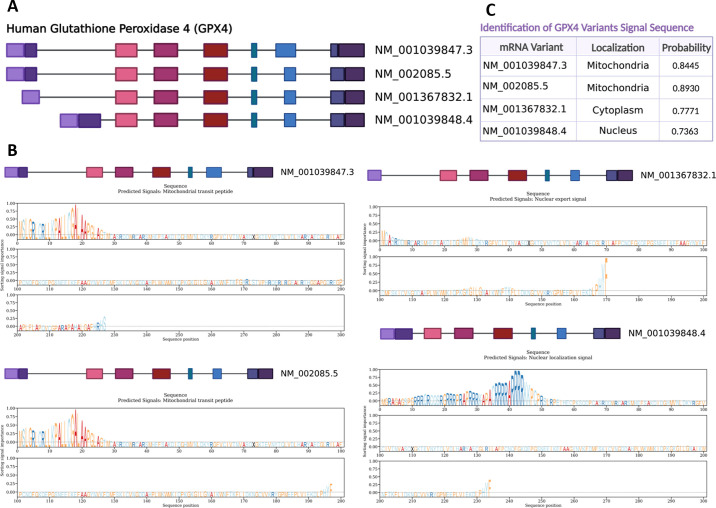
Human glutathione peroxidase 4 (GPX4) splice variants and signal sequence analysis. GPX4 splice variants were obtained from the National Center for Biotechnology Information (NCBI) database. The amino acid sequences were collected for each variant, and signal sequences were predicted using the DeepLoc-2.0 server. *A*: GPX4 splice variant schematic depicting the schematic representation of the GPX4 splice variants, highlighting structural exon and intron differences. *B*: GPX4 splice variant signal peptides and compartment showing the predicted signal peptides of the GPX4 splice variants, along with information about the cellular compartments to which they are targeted. *C*: predicted cellular compartment localization of GPX4 splice variants, along with the corresponding probability scores obtained from DeepLoc-2.0 server.

**Figure 4.**
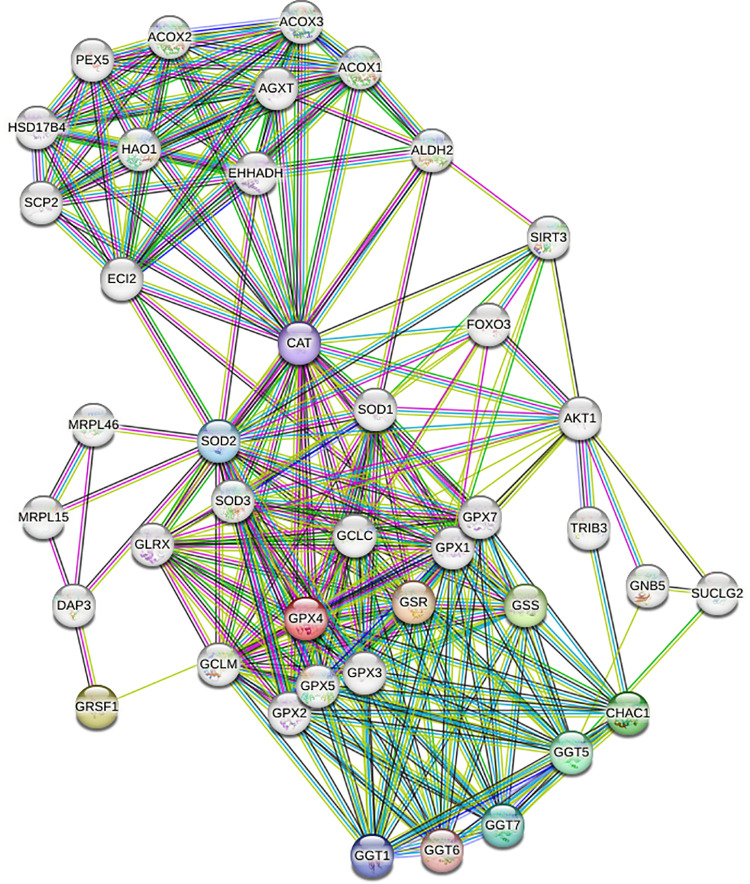
STRING protein-protein interaction (PPI) networks for human glutathione peroxidase 4 (GPX4). The PPI network for GPX4 was generated using the STRING database, incorporating known interactions from curated databases represented by light blue lines. Experimentally determined interactions are highlighted with magenta lines. Predicted interactions are color-coded in green, red, and blue. Yellow denotes text-mining. Black indicates coexpression. Light Blue signifies protein homology.

There are two established isoforms of GPX4: long and short. Mitochondrial GPX4 (mGPX4) is the short isoform of GPX4 with mitochondrial signal peptide at the NH_2_ terminus (108). We predicted a third form of GPX4 localized to the nucleus (Fig. 3). SLC25A39 imports mGPX4 and thus has a crucial role in regulating mitochondrial lipid peroxidation and consequently ferroptosis (109). GPX4 regulates multiple signaling molecules, which are important for DM-induced cardiac pathogenesis (Fig. 4). Thus, regulatory mechanisms underlying mGPX4 import and activity are important for mitochondrial ferroptosis.

Ferroptosis leads to mitochondrial damage, resulting in mitochondria that are smaller, crumpled, and fragmented (110). In RSL3-induced ferroptosis, mitochondria are fragmented and accumulated around the nucleus. Based on severity, mitochondria could be elongated (category 1), fragmented and uniformly distributed (category 2), and fragmented and mainly accumulated near the nucleus (category 3). MitoQ, the mitochondrial antioxidant, rescues cells from RSL3-induced mitochondrial damage (111). In erastin-induced ferroptosis in neuronal cells, transactivation of the BH3-interacting domain (BID) causes loss of mitochondrial membrane potential and promotes mitochondrial fragmentation, leading to reduced energy production (112).

Voltage-gated anion-selective channel 1 (VDAC1) is abundantly present on the outer membrane of mitochondria and regulates the flux of anionic hydrophilic metabolites, such as ATP, ADP, and respiratory substrates, between the cytosol and mitochondrial spaces (113, 114). There are three isoforms of VDACs: VDAC1, VDAC2, and VDAC3. Inhibition of all VDACs decreases mitochondrial membrane potential and thereby ATP generation. Mitochondrial metabolism through VDAC1 and VDAC2 is inhibited by free tubulin (114). Erastin binds to VDACs and antagonizes the inhibitory effect of free tubulin on VDACs, which leads to VDAC opening and mitochondrial hyperpolarization (115). In addition to hyperpolarization, the opening of VDAC also increases ROS and increases mitochondrial calcium overload, causing mitochondrial permeability transition pore opening that leads to the depletion of ATP (116, 117).

Despite the occurrence of mitochondrial damage in ferroptosis, there are counterintuitive findings on whether mitochondrial damage is a cause or a consequence of ferroptosis. Although mitochondrial-targeted suppression of lipid peroxidation inhibits ferroptosis, mitochondria-depleted cells can undergo ferroptosis, suggesting that mitochondria are not indispensable for ferroptosis (96, 118). Thus, it is posited that mitochondrial damage occurs at the late/terminal stage of ferroptosis and is a consequence of ferroptosis (96, 119).

Future investigations on mitochondrial mechanisms of ferroptosis in DMCM will provide novel insights and also new approaches to prevent and/or treat cardiac ferroptosis in different stages of DMCM.

## MISCONCEPTIONS ABOUT FERROPTOSIS

### Necrosis is Not Synonymous to Ferroptosis

Necrosis is a form of accidental cell death. The key feature of necrosis includes cytoplasmic swelling and plasma membrane rupture. In ferroptosis, these features are absent, at least in the initial stage of ferroptosis. Plasma membrane rupture could occur in the late stage of ferroptosis. The cause of cell membrane rupture is peroxidation of PUFA in the plasma membrane. An improved plasma membrane repair mechanism slows down cell death via ferroptosis (120). Furthermore, the propagation of ferroptosis between cells is independent of plasma membrane rupture, and rather, it depends on the release of oxidized lipids with intact plasma membrane, which is modulated by FSP1 and CoQ_10_ (87, 121). Erastin-induced ferroptosis exhibits increased mitochondrial membrane density and corresponding volume reduction, increased mitochondrial membrane potential, and swollen mitochondria (115, 119, 122). These features are absent in necrosis.

### Excess Lipid Does Not Always Drive Ferroptosis

Lipids, such as PUFA, are highly susceptible to ferroptosis. However, high levels of PUFA itself may not instigate ferroptosis because it requires ACSL4 and LPCAT3 for its activation and incorporation into the lipid membrane, respectively (5, 123). PUFA and mono-unsaturated fatty acids (MUFAs) have opposite effects on ferroptosis (124). PUFAs are proferroptosis, whereas MUFAs are antiferroptosis (125). Similar to PUFA and MUFA, the ACSLs associated with them have oppositive effects. ACSL4 activates PUFA and promotes ferroptosis, whereas ACSL3 activates MUFA to inhibit ferroptosis (125, 126). ACSL1, a member of the ACSL family, induces ferroptosis in some plant-based PUFA, such as α-eleostearic acid (127). Thus, all types of lipids do not drive ferroptosis, and even PUFA requires activation and membrane incorporation through ACSL4 and LPCAT3 to trigger ferroptosis.

### Oxidative Stress is Not Ferroptosis

ROS is an important signaling molecule to maintain cellular homeostasis. However, excess ROS causes oxidative stress (128). Excess ROS or oxidative stress per se does not induce ferroptosis, and ROS accumulation in the cell occurs without ferroptosis (1). Only lipid-ROS, as opposed to generalized ROS, are responsible for initiating lipid peroxidation, which in turn leads to ferroptosis. Antioxidants that specifically target lipid-ROS, such as GPX4, are uniquely capable of inhibiting this cell death pathway. Furthermore, ROS generated through the iron Fenton reaction plays a crucial role in driving ferroptosis, and this process can be mitigated by iron chelators like deferoxamine, which is an Food and Drug Administration (FDA)-approved drug (129).

## TARGETING FERROPTOSIS IN DIABETIC CARDIOMYOPATHY

**Figure 5.**
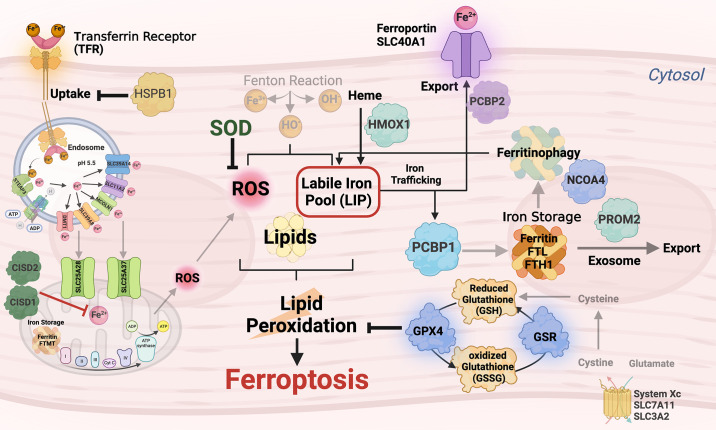
Targeting ferroptosis in diabetic cardiomyopathy. The key regulators of iron homeostasis, glutathione peroxidase 4 (GPX4) activity, and lipid peroxidation leading to ferroptosis in diabetic cardiomyopathy. Targeting the molecules that inhibit iron import, labile iron pool (LIP) formation, and lipid peroxidation have a high likelihood of mitigating diabetes-induced ferroptosis in cardiomyocytes. ADP, adenosine diphosphate; CISD1, CDGSH iron-sulfur domain 1; CISD2, CDGSH iron-sulfur domain 2; DMT1, divalent metal transporter 1; FTMT, mitochondria-specific form of ferritin; HMOX1; heme oxygenase-1; HSPB1, heat shock protein β-1; NCOA4, nuclear receptor coactivator 4; PCBP1, poly(rc)-binding protein 1; PCBP2, poly(rc)-binding protein 2; PROM2, prominin 2; ROS, reactive oxygen species; SOD, superoxide dismutase; STEAP3, six-transmembrane epithelial antigen of prostate 3. Figure created with BioRender.com.

The significance of ferroptosis in DMCM is an active area of research, and the underlying molecular mechanisms are still being investigated. Considering the essential role of ferroptosis in DMCM, targeting ferroptosis and iron metabolism may provide a novel therapeutic strategy to prevent and/or treat DMCM. Thus, further studies are warranted to completely understand the pathogenic mechanisms for diabetes-induced cardiac ferroptosis. The pathogenesis of DMCM is multifactorial; however, altered substrate metabolism and mitochondrial dysfunction are the major players leading to DMCM (130). Thus, targeting molecular mechanisms underpinning ferroptosis signaling in mitochondria, iron, and cysteine metabolisms are important to inhibit ferroptosis in DMCM (Fig. 5).

Diabetes-induced heart failure is distinct from nondiabetic heart failure in intramyocardial lipid accumulation and fatty acid oxidation (18, 131). The endoplasmic reticulum (ER) is the primary organelle for membrane lipid biosynthesis (132). The PUFA-PL clusters on the ER membrane and ER viscosity increase during ferroptosis, indicating that the volume of ER may determine the sensitivity to ferroptosis (53, 133). Suppressing ER stress through dapagliflozin, a sodium-glucose cotransporter 2 inhibitor, improves subclinical myocardial function in diabetes (Dapagliflozin and Prevention of Adverse Outcomes in Heart Failure trial) (134). ER membrane also communicates to mitochondria via mitochondria-associated ER membranes (MAMs) to regulate lipid and metabolite exchange. Diabetes disrupts the structure and function of MAMs (135). The stimulated Raman scattering microscopy revealed that ferrostatins (including ferrostatin-1) accumulate in the ER and mitochondria (136). These findings suggest that ER, mitochondrial, and MAMs could be promising subcellular targets to inhibit ferroptosis in DMCM.

The higher risk of heart failure in females with diabetes is plausibly due to lower number of mitochondria in cardiomyocytes and higher myocardial steatosis (131). Ferroptosis process differs between males and females (137). Thus, it is important to consider sex as a biological variable while investigating and targeting ferroptosis in DMCM.

Because of altered levels of insulin, the T1DM heart differs from the T2DM heart in pathogenesis and molecular signaling (131, 138, 139). The mammalian target of rapamycin (mTOR), a serine/threonine kinase, and its complexes mTOR complex 1 (mTORC1) and mTORC2 are key regulators of insulin signaling (140). mTORC1 activates insulin receptor substrate 1 for downstream insulin signaling (141). Cysteine activates mTORC1 to induce GPX4 expression, and pharmacological inhibition of mTORC1 downregulates GPX4 protein synthesis (142). Thus, it is important to consider the type of diabetes while investigating and/or targeting ferroptosis in DMCM. The levels of lipid accumulation in the cardiomyocytes are associated with pathogenesis and cardiac dysfunction in patients with diabetes (18). Thus, the stage of diabetes, early versus advanced, should be considered while investigating and targeting ferroptosis in DMCM.

As PUFA is the substrate for lipid peroxidation, reducing the levels of PUFA may be an approach to prevent and/or treat cardiac ferroptosis in DMCM. Redox-active iron (LIP) is indispensable for ferroptosis. Thus, iron chelators may be a therapeutic option to mitigate cardiac ferroptosis in DMCM. Mitochondria is involved in ROS production and several cell death mechanisms. Thus, mitochondrial-mediated other cell death mechanisms may contribute to ferroptosis. Antioxidants may aid in preventing lipid peroxidation via autophagy machinery (143).

## FUTURE DIRECTIONS

It has been shown that hypoglycemic medicine sodium-glucose cotransporter type-2 inhibitors (SGLT2i) affect iron homeostasis, and thereby may have potential cardioprotective effects via suppressing ferroptosis in DMCM (144). The glucose-lowering medication rosiglitazone inhibits ACSL4, thereby reducing lipid peroxidation and iron content in renal tubular cells, which helps mitigate diabetic nephropathy (145). Thus, preventing and/or treating cardiac ferroptosis has the potential to mitigate DMCM. Molecular mechanisms underpinning ferroptosis are beginning to unfold in DMCM (7, 8, 146, 147). DM is a complex disease that affects multiple signaling pathways. Thus, it is important to investigate direct and indirect molecular signaling pathways regulating ferroptosis in DMCM (1, 17, 51, 148). Mitochondrial dysfunction is a hallmark in both T1DM and T2DM hearts (149). Thus, studies involving the association of mitochondrial dysfunction with ferroptosis may unravel new regulatory mechanisms of ferroptosis in DMCM.

Although NRF2-mediated regulation of iron storage and export has been studied in the DM heart, more studies on iron import mechanisms and other uncovered iron export mechanisms are warranted to understand iron homeostasis, and, thereby, regulation of ferroptosis in DMCM (9, 45). Iron is also imported and stored inside mitochondria, and the trafficking of iron between mitochondria and cytoplasm has a crucial role in iron homeostasis in the cell (99). There is a gap in knowledge on how diabetes influences iron trafficking between mitochondria and cytoplasm. Future studies on molecular mechanisms regulating iron trafficking between mitochondrial and cytoplasm may open a new window for mitochondrial iron homeostasis in the DM heart and in DMCM. Ferritinophagy, the autophagy of ferritin that produces LIP, is important for iron metabolism and ferroptosis (150). Autophagy is differentially regulated between T1DM and T2DM hearts (138). Investigations on diabetes-induced regulation of myocardial ferritinophagy may delineate the regulation of iron homeostasis between T1DM and T2DM hearts.

AMPK is a key player in the regulation of autophagy. It blocks acyl CoA formation, which is a precursor of PUFA (1, 151). Future studies on the role of ER in the formation of PUFA and communication between ER and mitochondria in DMCM are warranted. Research into the regulation of PUFA formation and its role in membrane lipid peroxidation in the DM heart will deepen our understanding of both lipid peroxidation process and ferroptosis regulation in DMCM.

Hydrogen sulfide (H_2_S) is downregulated in the DM heart, and H_2_S donor is cardioprotective and prevents DMCM (19, 152, 153). H_2_S donor treatment increases SLC7a11 in hyperglycemic myotubes, and SCL7a11 regulates cysteine import in the T2DM heart (9, 154). Cysteine is the rate-limiting metabolite of glutathione biosynthesis, which is required for GPX4 activity (155). Furthermore, H_2_S regulates sulfur metabolites (156). One possibility is that H_2_S may be involved in iron-sulfur clustering in mitochondria. Future studies on the roles of H_2_S signaling in ferroptosis may reveal novel regulatory pathways for ferroptosis in DMCM.

Drugs that affect other cell death mechanisms may also affect ferroptosis. For example, dexmedetomidine, which inhibits apoptosis in the DM heart, is reported to attenuate ferroptosis through the Nrf2/GPX4 pathway in hyperglycemic cardiomyocytes (H9C2 cells) (157). Although the link between ferroptosis and other forms of cardiac cell death is still under investigation, it is plausible that ferroptosis mechanism may be linked to other cell death mechanisms, such as necroptosis, apoptosis, and pyroptosis (158).

Although antioxidants are not directly involved in inhibiting ferroptosis in DMCM, scavengers and inhibitors of lipid peroxides, such as ferrostatin-1 and liprostatin-1, play crucial roles in ferroptosis inhibition (159). Small molecules that inhibit selenoprotein GPX4, such as RSL3, ML162, and ML210, have been successfully used to induce cardiac ferroptosis. These small molecule inhibitors are valuable for investigating the contribution of ferroptosis in myocardial cell death in DMCM. Ferrostatin-1, however, suppresses the effects of these small molecules. Thioredoxin reductase 1 (TXNRD1) is a newly discovered selenoprotein. TRi-1 and TRi-2 are compounds that inhibit TXNRD1, and cells treated with these compounds are not rescued by ferrostatin-1 (160). These molecules are valuable for revealing novel regulatory mechanisms of ferroptosis in DMCM.

### Perspectives and Significance

**Figure 6.**
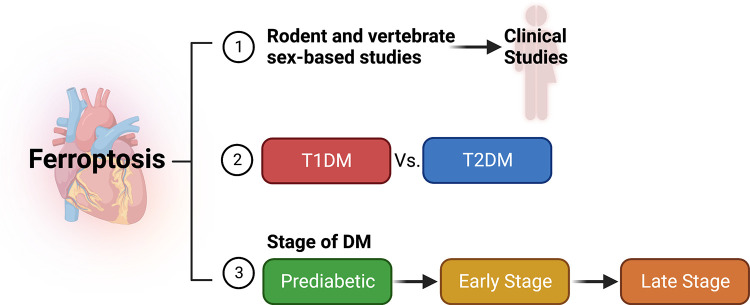
Summary of future direction for ferroptosis studies in diabetic cardiomyopathy. An overview of potential future research directions and key aspects related to the investigation of ferroptosis in the context of the diabetic heart. *1*: considering sex as a biological variable, there is a need to conduct sex-based studies on ferroptosis in rodent and vertebrate animal models, as well as human clinical studies to determine the role of ferroptosis in sex-specific diabetic cardiomyopathy. *2*: considering type of diabetes, there is a need to compare the molecular mechanisms of ferroptosis in type 1 diabetes mellitus (T1DM) and type 2 diabetes mellitus (T2DM) to determine the differences in their ferroptosis mechanisms. *3*: considering the stage of diabetes, the pathophysiology and pathogenesis in the heart varies with different stages of diabetes, particularly pre-, early-, and late-stage diabetes. Thus, it is important to examine ferroptosis during these stages. Figure created with BioRender.com.

In summary, we have a little understanding of the regulation of ferroptosis in DMCM. We may use the wealth of knowledge on ferroptosis mechanisms and its regulation in other diseases and models in a context-dependent manner to unravel the comprehensive regulatory mechanisms of ferroptosis in DMCM. As ferroptosis is influenced by the sex, pathophysiological conditions, and types of diabetes, it is important that studies targeting diabetes-induced myocardial ferroptosis must include sex, type of diabetes (T1DM or T2DM), and stage of diabetes (prediabetes, early, or late diabetes) (Fig. 6).

## GRANTS

We acknowledge funding supports from the National Institutes of Health Grant R56HL156806 and the University of Nebraska Collaboration Initiative grants to P.K.M. and the UNMC Presidential Graduate Fellowship to F.I.G.

## DISCLOSURES

No conflicts of interest, financial or otherwise, are declared by the authors.

## AUTHOR CONTRIBUTIONS

P.K.M. conceived and designed research; F.I.G. prepared figures; P.K.M. drafted manuscript; F.I.G. and P.K.M. edited and revised manuscript; F.I.G. and P.K.M. approved final version of manuscript.
